# Technique modifications: enabling laparoscopic repair of duodenal atresia in a preterm, very low birthweight infant

**DOI:** 10.1093/jscr/rjaf323

**Published:** 2025-05-30

**Authors:** Patricia Corujo Avila, John M Woodward, Joseph C L’Huillier, Brie Mucci-Jackson, Ruchi Amin, Mark L Wulkan, P Benson Ham 3rd

**Affiliations:** Jacobs School of Medicine and Biomedical Sciences, University at Buffalo, State University of New York, 100 High Street, Buffalo, NY 14203, United States; Division of Pediatric Surgery, John R. Oishei Children’s Hospital, 1001 Main Street, Buffalo, NY 14203, United States; Department of Surgery, University at Buffalo, Jacobs School of Medicine and Biomedical Sciences, 100 High Street, Buffalo, NY 14203, United States; Division of Pediatric Surgery, John R. Oishei Children’s Hospital, 1001 Main Street, Buffalo, NY 14203, United States; Department of Surgery, University at Buffalo, Jacobs School of Medicine and Biomedical Sciences, 100 High Street, Buffalo, NY 14203, United States; Division of Pediatric Surgery, John R. Oishei Children’s Hospital, 1001 Main Street, Buffalo, NY 14203, United States; Department of Surgery, University at Buffalo, Jacobs School of Medicine and Biomedical Sciences, 100 High Street, Buffalo, NY 14203, United States; Division of Pediatric Surgery, John R. Oishei Children’s Hospital, 1001 Main Street, Buffalo, NY 14203, United States; Department of Surgery, University at Buffalo, Jacobs School of Medicine and Biomedical Sciences, 100 High Street, Buffalo, NY 14203, United States; Division of Pediatric Surgery, Arthur M. Blank Hospital, Children’s Healthcare of Atlanta, 2174 North Druid Hills Road NE 1st Floor, Atlanta, GA 30329, United States; Division of Pediatric Surgery, Department of Surgery, Emory University School of Medicine, 100 Woodruff Circle, Atlanta, GA 30322 United States; Division of Pediatric Surgery, John R. Oishei Children’s Hospital, 1001 Main Street, Buffalo, NY 14203, United States; Department of Surgery, University at Buffalo, Jacobs School of Medicine and Biomedical Sciences, 100 High Street, Buffalo, NY 14203, United States

**Keywords:** duodenal atresia, very-low-birth-weight infant, preterm, duodenoduodenostomy

## Abstract

Laparoscopic procedures, which are already challenging in infants and small children, are made even more challenging in very low birthweight infants due to the limited working space within the abdomen and the decreased tolerance for high insufflation pressures. Here we describe how modifying port placements and instrument positioning allowed for the laparoscopic repair of duodenal atresia in a preterm 1.3 kg infant. In our modified approach: (i) the umbilical-port was our right-hand working port, (ii) the right lower quadrant port was used for the telescope, (iii) the left central abdomen port was used as an optional assistant port, and (iv) the right upper quadrant port, was modified to be in the right lateral upper abdomen and used as the left hand working port. We believe these modifications could help pediatric surgeons maximize laparoscopic working space and therefore, prevent complications and improve patient outcomes for the procedure.

## Introduction

Duodenal atresia is the most common cause of congenital small intestine atresia and occurs in approximately one infant per 10 000 births [1]. Surgical repair can be done open or laparoscopic, with the first laparoscopic repairs reported by Bax *et al*. [2] and Rothenberg [3] in the early 2000s. While a challenging procedure, the literature has shown that patients who underwent laparoscopic repair benefit from quicker recovery times and shorter times to full oral feeds compared with the traditional, open approach [4]. We reviewed the literature for published techniques in very low birthweight infants; however, we found limited strategies to help facilitate success. Here, we describe how we modified the port placements and instrument positioning during a duodenoduodenostomy to maximize working space in a very low birthweight, preterm infant, and facilitate laparoscopic repair.

## Case report

**Figure 1:**
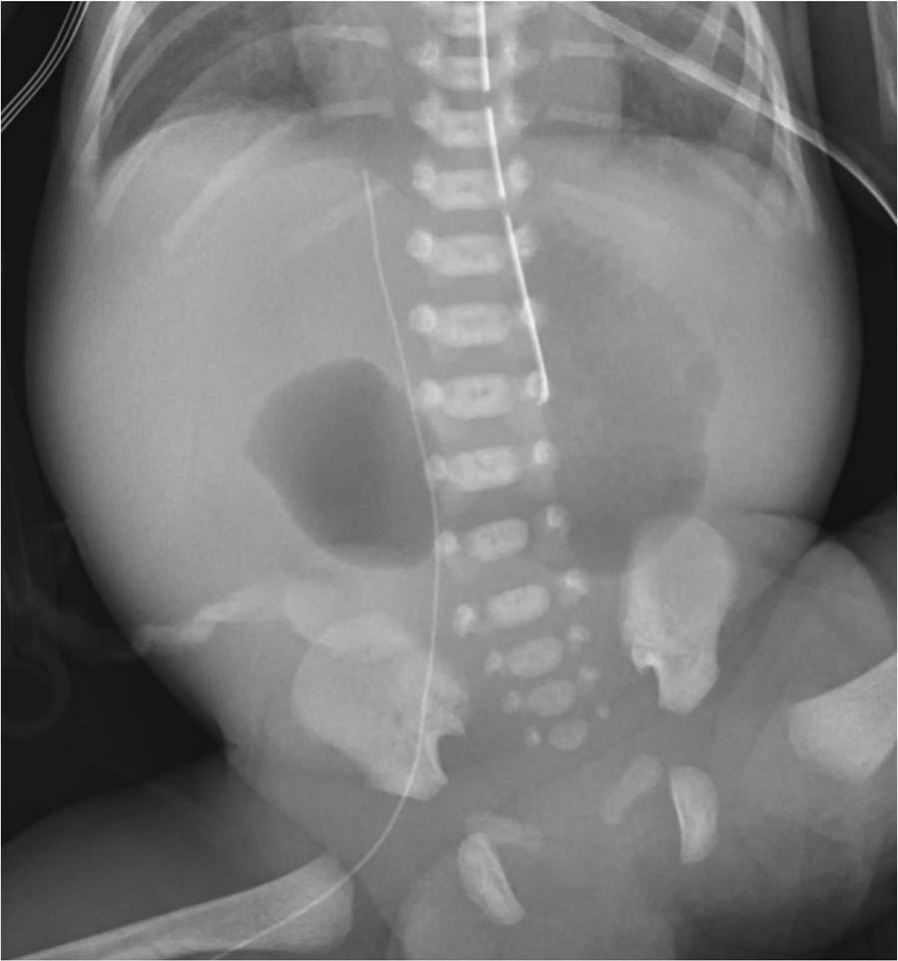
“Double bubble” sign on X-ray.

A male was born at 30 weeks via cesarean-section to a 37-year-old mother whose pregnancy was complicated by maternal obesity, polyhydramnios requiring amnioreduction, positive Group B Streptococcus (GBS) screen, and a maternal enterococcal urinary tract infection (UTI) at birth. This patient had appearance, pulse, grimace, activity, and respiration (APGAR) scores of 4 and 7, at 1 and 5 min, respectively. Duodenal atresia was suspected prenatally as a “double bubble” sign had been observed on fetal ultrasound. The diagnosis was confirmed after birth with an abdominal radiograph redemonstrating a double bubble sign (Fig. 1). The patient was admitted to the neonatal intensive care unit (NICU) with a supplemental oxygen requirement and orogastric tube (OGT). On day of life (DOL) two, the patient developed bacteremia with gram positive cocci in pairs and chains. Infectious Disease recommended treating with a 2-week course of ampicillin and gentamicin. The NICU advocated for delaying surgery, so the patient was maintained NPO, receiving total parenteral nutrition (TPN) with lipids via a peripherally inserted central catheter (PICC) line. On DOL seven, blood cultures were negative, the patient weighted 1300 g, and was determined ready for surgery.

**Figure 2:**
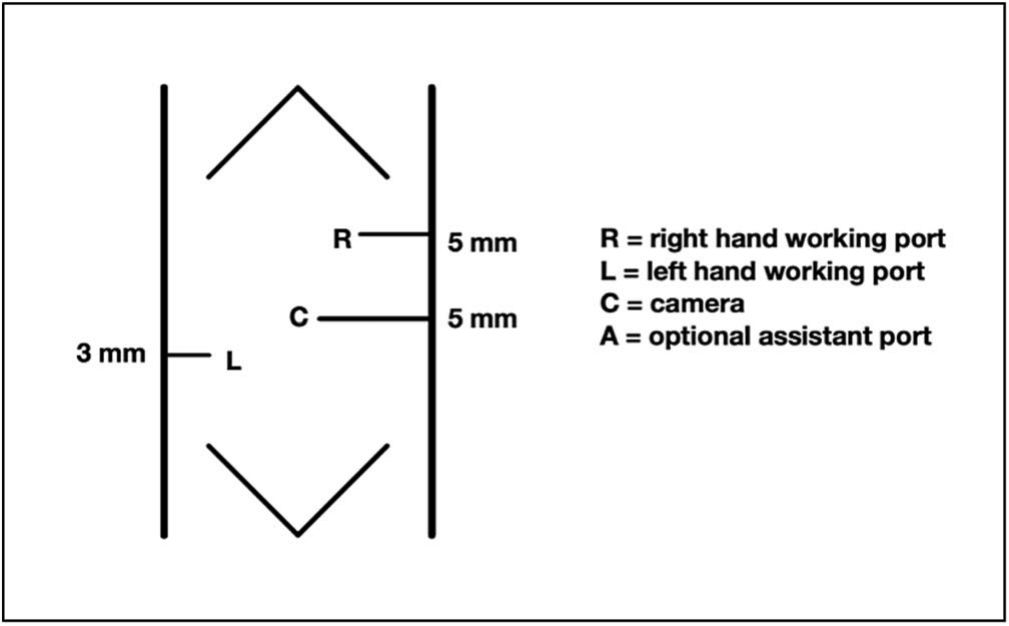
Standard laparoscopic duodenostomy port placement.

**Figure 3:**
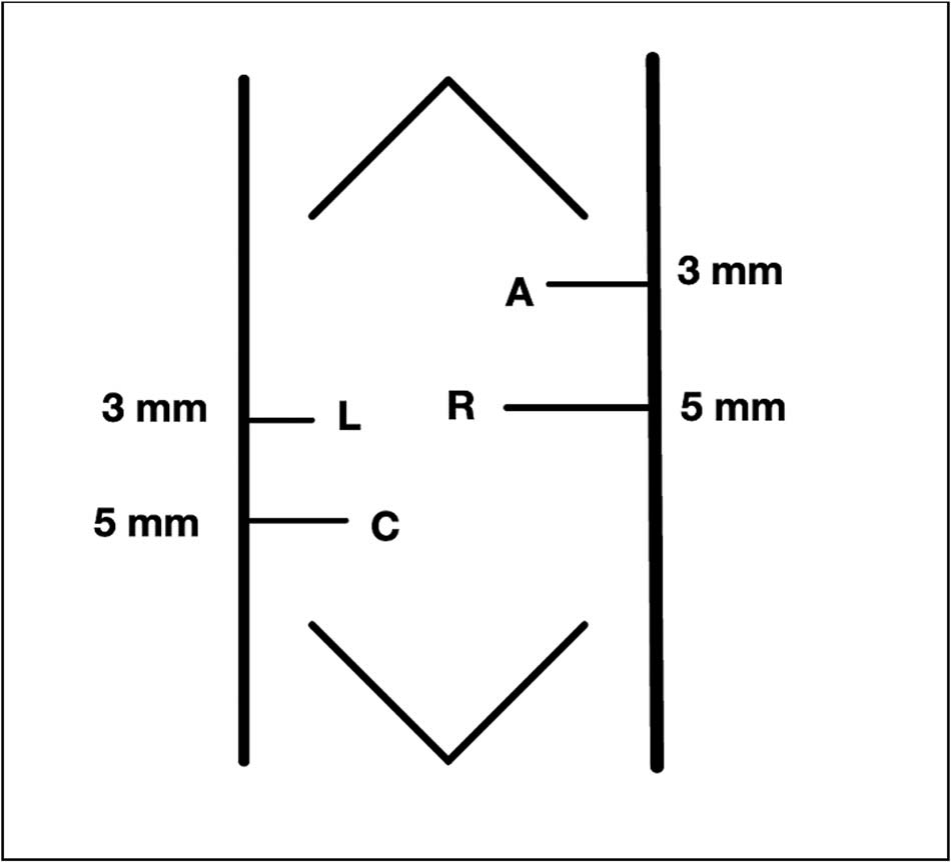
Our modified port placement.

To maximize the working space available in this very low birth weight infant, we modified the traditional laparoscopic port placement for duodenoduodenostomies (Fig. 2). The first port was placed through the umbilicus, followed by three additional ports as shown in Fig. 3: one in the right lower quadrant, one in the right lateral upper quadrant, and one in the left central abdomen. The patient tolerated 8 mm Hg of insufflation. In our modified approach: (i) the umbilical-port, traditionally used for the surgical telescope was our right-hand working port, (ii) the right lower quadrant port was used for the telescope, (iii) the left central abdomen port, typically the surgeons right-hand working port, was used as an optional assistant port if needed, and (iv) the right upper quadrant port, was modified to be in the right lateral upper abdomen and used as the left hand working port.

Intraoperatively, the stomach and proximal duodenum were dilated. The gallbladder was large, distended, and densely adherent to the transverse colon. The cecum was in the right middle abdomen with the appendix flipped up toward the gallbladder, attached to the right upper quadrant through adhesions. After taking down the adhesions with hook cautery and dissecting the structures obscuring the field of view for the anastomosis, the pancreas and distal duodenum were visualized. The duodenum was kocherized and the distal duodenum mobilized. A 1.2 cm transverse enterotomy was made in the duodenum, ⁓1 cm proximal to the obstruction, and a similar size longitudinal enterotomy was made distally. A 4–0 PDS percutaneous suture was placed as the first stitch and transfixed over a gauze bolster to elevate the anastomosis and line it up. A duodenoduodenostomy was created by running the posterior anastomotic wall with 4–0 Vicryl cut to 12 cm and ended by tying to itself, and the same was done with another 4–0 Vicryl to run the front wall. The needle tip was bent by the port halfway through the anterior wall as the interior working space was very limited in this tiny premature infant, so that suture was tied to itself, and the rest of the anterior wall was finished with interrupted sutures.

Post-operatively, the patient was returned to the NICU and recovered well. The patient was extubated on post-op day two (POD #2), and an OGT was left in place post-operatively until POD #7 when it was removed, and feeds were started. Feeds were advanced and the patient was able to be discharged on full oral feeds 1 month after surgery.

## Discussion

The laparoscopic repair of duodenal atresia in very low birthweight infants is made more challenging than other laparoscopic surgeries for various reasons: the patients’ size limits the surgeon’s working space, and the delicate nature of the patients’ condition leaves little margin for error. The American Pediatric Surgeons association online library “Not a Textbook” section on duodenal atresia repair states, “Children less than 1.5 kg in weight may lack the working volume required to complete intracorporeal knot tying” [5]. Furthermore, a 2022 study found that early preterm neonates and specifically those with very low birthweight, were significantly more likely to experience postoperative complications and a prolonged time to full oral intake after surgical repair of duodenal atresia [6].

**Figure 4:**
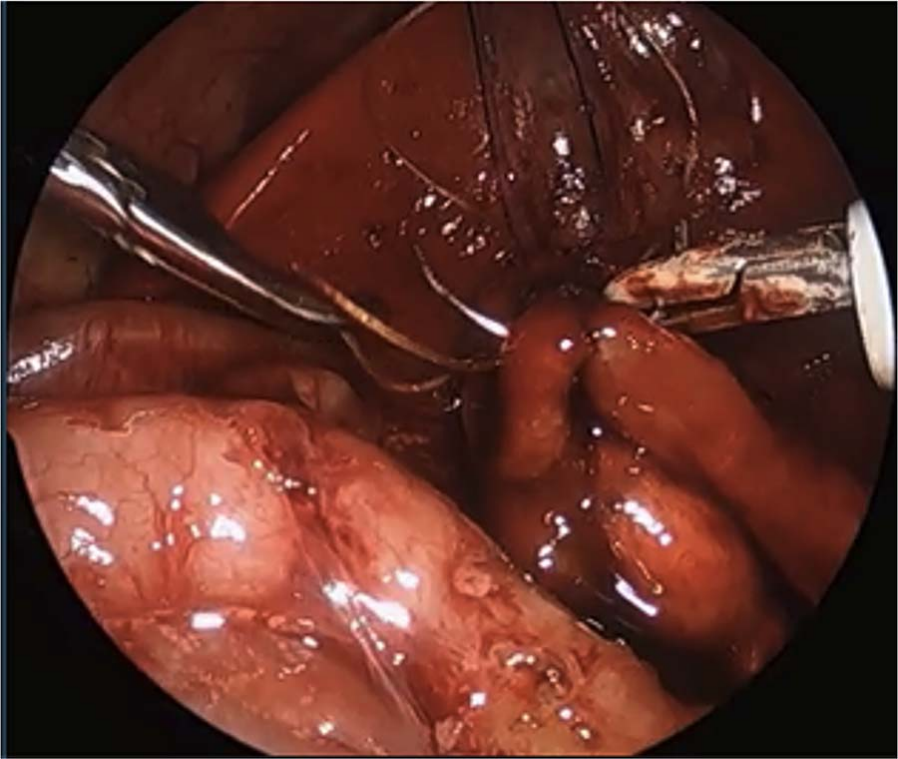
Laparoscopic view of anastomosis. The surgery was completed successfully laparoscopically, and the patient had no postoperative complications.

We describe a modification of the port placements during a laparoscopic duodenal atresia repair in a very low birthweight, preterm infant; however, these modifications can be used to optimize repair in any duodenal atresia patient. The modified positioning of the instruments allowed for a working space better triangulated for suturing with both hands at right angles to each other and allowed for the camera to be further away from the target tissue. These modified placements also enabled a more lateral approach, more in line with the rotation of the duodenum, and facilitated easier angles for laparoscopic suturing with a straight-on view of the anastomosis, overall increasing the safety of the procedure and limiting the risk of intraoperative complications (Fig. 4).

Additionally, during the operation the distal duodenum had a normal course with no malrotation. If we had identified malrotation we would have performed a concurrent Ladd’s procedure, which could be performed with the modified port placement, and would improve visualization and exposure for the duodenoduodenostomy.

In conclusion, we believe modified port placement and alternative instrument positioning in laparoscopic duodenoduodenostomy facilitates repair of duodenal atresia in very low birthweight infants and should be considered for all duodenal atresia patients due to the better visualization and suturing angles provided by this technique.

## Prior communication

This is an original work that has not been published and is not under consideration at any other journals.
